# Phenotypic Variation in the Group A *Streptococcus* Due to Natural Mutation of the Accessory Protein-Encoding Gene *rocA*

**DOI:** 10.1128/mSphere.00519-18

**Published:** 2018-10-17

**Authors:** Poulomee Sarkar, Jessica L. Danger, Ira Jain, Laura A. Meadows, Christopher Beam, Josette Medicielo, Cameron Burgess, James M. Musser, Paul Sumby

**Affiliations:** a Department of Microbiology & Immunology, University of Nevada, Reno School of Medicine, Reno, Nevada, USA; b Center for Molecular and Translational Human Infectious Diseases Research, Houston Methodist Research Institute, Houston, Texas, USA; c Department of Pathology and Genomic Medicine, Houston Methodist Hospital, Houston, Texas, USA; University of Iowa

**Keywords:** *Streptococcus pyogenes*, gene mutation, gene regulation, phenotypic variation

## Abstract

Populations of a bacterial pathogen, whether recovered from a single patient or from a worldwide study, are often a heterogeneous mix of genetically and phenotypically divergent strains. Such heterogeneity is of value in changing environments and arises via mechanisms such as gene gain or gene mutation. Here, we identify an isolate of serotype M12 group A *Streptococcus* (GAS) (Streptococcus pyogenes) that has a natural mutation in *rocA*, which encodes an accessory protein to the virulence-regulating two-component system CovR/CovS (CovR/S). Disruption of RocA activity results in the differential expression of multiple GAS virulence factors, including the anti-phagocytic hyaluronic acid capsule and the chemokine protease SpyCEP. While some of our data regarding RocA-regulated genes overlaps with previous studies, which were performed with isolates of alternate GAS serotypes, some variability was also observed. Perhaps as a consequence of this alternate regulatory activity, we discovered that the contribution of RocA to the ability of the M12 isolate to survive and proliferate in human blood *ex vivo* is opposite that previously observed in M1, M3, and M18 GAS strains. Specifically, *rocA* mutation reduced, rather than enhanced, survival of the isolate. Finally, we also present data from an analysis of *rocA* transcription and show that *rocA* is transcribed in both mono- and polycistronic mRNAs. In aggregate, our data provide insight into the important regulatory role of RocA and into the mechanisms and consequences of GAS phenotypic heterogeneity.

**IMPORTANCE** This study investigates the regulatory and phenotypic consequences of a naturally occurring mutation in a strain of the bacterial pathogen the group A *Streptococcus* (Streptococcus pyogenes). We show that this mutation, which occurs in a regulator-encoding gene, *rocA*, leads to altered virulence factor expression and reduces the ability of this isolate to survive in human blood. Critically, the blood survival phenotype and the assortment of genes regulated by RocA differ compared to previous studies into RocA activity. The data are consistent with there being strain- or serotype-specific variability in RocA function. Given that phenotypic variants can lead to treatment failures and escape from preventative regimes, our data provide information with regard to a mechanism of phenotypic variation in a prevalent Gram-positive pathogen.

## Abstract (precis)

This study investigates the regulatory and phenotypic consequences of a naturally occurring mutation in a strain of the bacterial pathogen the group A *Streptococcus* (Streptococcus pyogenes). We show that this mutation, which occurs in a regulator-encoding gene, *rocA*, leads to altered virulence factor expression and reduces the ability of this isolate to survive in human blood. Critically, the blood survival phenotype and the assortment of genes regulated by RocA differ compared to previous studies into RocA activity. The data are consistent with there being strain- or serotype-specific variability in RocA function. Given that phenotypic variants can lead to treatment failures and escape from preventative regimes, our data provide information with regard to a mechanism of phenotypic variation in a prevalent Gram-positive pathogen.

## INTRODUCTION

Phenotypic heterogeneity among isolates of a given bacterial species is a commonly observed phenomenon. As examples, isolates of the food-borne pathogen Listeria monocytogenes can be variable in their growth and virulence (1, 2), while isolates of Enterococcus faecium can be variable with regard to their antibiotic resistance profiles (3). Mechanisms driving such phenotypic heterogeneity include gene gain, gene loss, gene mutation, recombination events, epigenetic changes, and phase variation mechanisms (4).

The group A *Streptococcus* (GAS) (Streptococcus pyogenes) is a bacterial pathogen that is capable of causing distinct diseases in humans from mild and self-limiting pharyngitis (also known as strep throat) to severe and life-threatening necrotizing fasciitis (also known as the flesh-eating infection) (5). GAS strains are divided into serotypes based upon the sequence of the 5′ end of the *emm* gene, a gene that encodes the classical GAS virulence factor the M protein (6). We and others have characterized serotype-specific variation in GAS disease potential, with certain serotypes being nonrandomly associated with particular disease manifestations (7–9). For example, serotype M3 isolates are nonrandomly associated with cases of necrotizing fasciitis, and serotype M18 isolates are nonrandomly associated with cases of acute rheumatic fever (10, 11).

Strain-specific variation has also been described for GAS, with differences in the number of tandem repeat sequences in DNA being a major mechanism of strain-specific genetic, and subsequently phenotypic, variation. Some variable number tandem repeats (VNTRs) have been described within intergenic regions, where additions or deletions in the number of repeats control transcription of the downstream gene (12, 13). In other cases, VNTRs are located within genes where changes in the number of repeats can result in the expression of antigenic variants or in the introduction of premature stop codons (14–18). In addition to dividing VNTRs along intra- and intergenic lines, they can also be divided by the type of gene affected. For example, some are located within select genes that encode cell surface proteins, consistent with VNTR variation in these genes being a mechanism to introduce antigenic variation and circumvent antibody-mediated killing (16, 17). Perhaps the largest class of genes that harbor identified VNTRs are those that encode regulators of gene transcription, with certain VNTR lengths enabling, and others preventing, the activity of the regulatory protein (10, 11, 14, 15).

The control of virulence (Cov) two-component regulatory system (also known as Csr) controls the abundance of more than 10% of GAS gene transcripts, including many that encode immunomodulatory virulence factors (19–25). CovS is a membrane-spanning sensor kinase that modifies the phosphorylation status of CovR, a cytoplasmically located response regulator (26–28). Activated (phosphorylated) CovR primarily functions as a repressor protein, modulating both the assortment and abundance of gene transcripts. CovR/CovS (CovR/S) are key regulators of GAS disease potential. This is perhaps best exemplified by the fact that more than 15% of GAS isolates recovered from invasive infections have mutations in *covR* and/or *covS* (29–31). Multiple studies have identified that such strain-specific mutant derivatives are positively selected for during invasive infections, with enhanced protection against neutrophil-mediated killing being a major phenotype associated with *covR* or *covS* mutation (14, 23, 32–34). We recently identified that the regulator of cov (RocA) protein functions as an accessory protein to the CovR/S system, such that there is only minimal activity to this system in the absence of RocA (35). How RocA enhances CovR/S activity is under investigation. While not as well studied, or as prevalent, as *covR* or *covS* mutant GAS strains, clinical isolates with mutations in *rocA* have been described (34, 36, 37). Here, we identify and characterize a naturally occurring *rocA* mutant strain of serotype M12 GAS. We show that the *rocA* mutation dramatically alters virulence factor expression and that this differential regulation alters the ability of GAS to survive and proliferate in human blood. However, unlike previous studies performed in serotype M1, M3, and M18 GAS strains (11, 35, 38), our work in the M12 background identified that *rocA* mutation reduced, rather than enhanced, survival in blood *ex vivo*. We also characterize transcription of the *rocA* gene and show that in addition to being transcribed from its own promoter, it is also cotranscribed with an upstream tRNA methyltransferase-encoding gene. *In toto*, the data enhance our understanding of the mechanisms driving phenotypic variation in a prevalent Gram-positive bacterial pathogen.

## RESULTS

### Differing *rocA* alleles in two serotype M12 GAS isolates.

**FIG 1:**
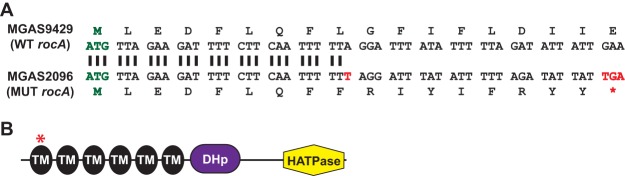
The *rocA* mutation in strain MGAS2096 results in the early truncation of the protein. (A) Comparison of a section of the *rocA* gene and of the translated product between the serotype M12 isolates MGAS2096 and MGAS9429. The start codons are shown in green. The location of the 1-bp insert in MGAS2096 is highlighted in red, as is the location of the stop codon that is subsequently introduced. WT, wild type; MUT, mutant. (B) Domain structure of the RocA protein. The location of the truncation in strain MGAS2096 is highlighted with a red asterisk. RocA has six putative transmembrane domains (TM) (black), a putative dimerization and histidine phosphotransfer domain (DHp; purple), and a putative histidine kinase-like catalytic domain (HATPase; yellow).

The whole-genome sequences of two serotype M12 isolates, MGAS2096 and MGAS9429, were published in 2007 (39). MGAS2096 was isolated from a patient with acute poststreptococcal glomerulonephritis (APSGN) in Trinidad in 1960. This organism, also known as strain A374, has been studied previously (40, 41). MGAS9429 was cultured from a pediatric patient with pharyngitis in Texas in 2001. Despite being isolated 41 years apart and from patients in different countries, the core genomes of MGAS2096 and MGAS9429 differ by only 290 single nucleotide polymorphisms (18). As part of our efforts to characterize the activity of the accessory protein RocA, we found that strains MGAS2096 and MGAS9429 have different *rocA* alleles. Specifically, the *rocA* allele in MGAS2096 has a one base pair insertion within the 5′ end of the gene, which results in the formation of a premature stop codon (Fig. 1A). The truncated RocA protein produced by this strain is only 17 amino acids in size, compared to 451 amino acids for the full-length protein (Fig. 1B). Thus, the sequence data suggest that MGAS2096 has a null mutant *rocA* allele.

### Regulatory and phenotypic variability between MGAS2096 and MGAS9429 isolates.

**FIG 2:**
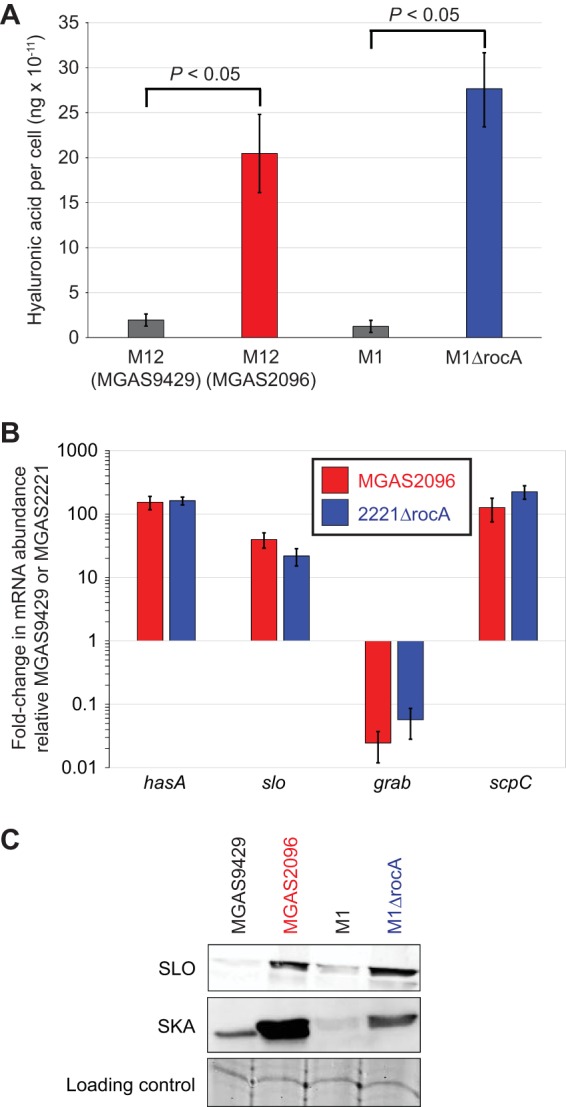
Regulatory and phenotypic differences between the clinical M12 isolates MGAS9429 and MGAS2096 mirror those between the clinical M1 isolate MGAS2221 and its isogenic *rocA* mutant derivative. (A) Assay of capsule expression. Exponential-phase cultures of the indicated GAS strains were analyzed for levels of the hyaluronic acid capsule. The experiment was performed on three occasions, using duplicate cultures of each strain in each experiment, and the values shown are means ± standard deviations (error bars). Statistical significance was determined by the Wilcoxon signed rank test (*P  < *0.05). (B) TaqMan-based quantitative RT-PCR analysis. Shown is the fold change in abundance of select mRNAs in strain MGAS2096 relative to MGAS9429 and in strain 2221ΔrocA relative to MGAS2221. Values are averages ± standard deviations (error bars) for duplicate samples run in triplicate. All data points shown are statistically significant (*P < *0.05 by Wilcoxon signed rank test). (C) Western blot analyses comparing expression of the secreted GAS proteins streptolysin O (SLO) and streptokinase (SKA). A representative band from a Coomassie blue-stained gel is shown as a loading control.

If MGAS2096, but not MGAS9429, has a null mutant *rocA* allele, then we hypothesized that we would be able to identify phenotypic differences between these isolates. The first phenotype we tested was the level of hyaluronic acid capsule expression, as expression of the anti-phagocytic capsule is highly repressed by the CovR/S system in the presence, but not in the absence, of functional RocA (11, 38, 42). The hyaluronic acid capsule was 10-fold more abundant in strain MGAS2096 than in strain MGAS9429 (Fig. 2A), following a similar pattern to that previously observed between the serotype M1 strain MGAS2221 (M1) and its constructed *rocA* mutant derivative M1ΔrocA (Fig. 2A). We next compared transcript levels between strain pair MGAS2096 and MGAS9429 and strain pair MGAS2221 and 2221ΔrocA, from several CovR/S-regulated virulence factor-encoding genes. Relative to MGAS9429, MGAS2096 had significantly higher levels of mRNAs from the *hasA* (hyaluronan synthase; involved in capsule biosynthesis [43]), *slo* (streptolysin O, a hemolysin [44]), and *scpC* (SpyCEP, a chemokine protease [45]) genes, and lower levels of mRNA from the *grab* (protein G-related α2-macroglobulin-binding protein; a protease inhibitor-binding protein [46]) gene (Fig. 2B). This regulatory pattern is essentially identical to that gained by comparing strains MGAS2221 and 2221ΔrocA (Fig. 2B), consistent with the regulatory and phenotypic differences observed between the two M12 strains being at least in part a consequence of the *rocA* mutation in MGAS2096. This conclusion was supported by Western blot data showing that the levels of expression of streptolysin O (SLO) and streptokinase (SKA, a thrombolytic factor [47]) differed (Fig. 2C).

### The *rocA* mutation in strain MGAS2096 can be complemented by a wild-type allele.

**FIG 3:**
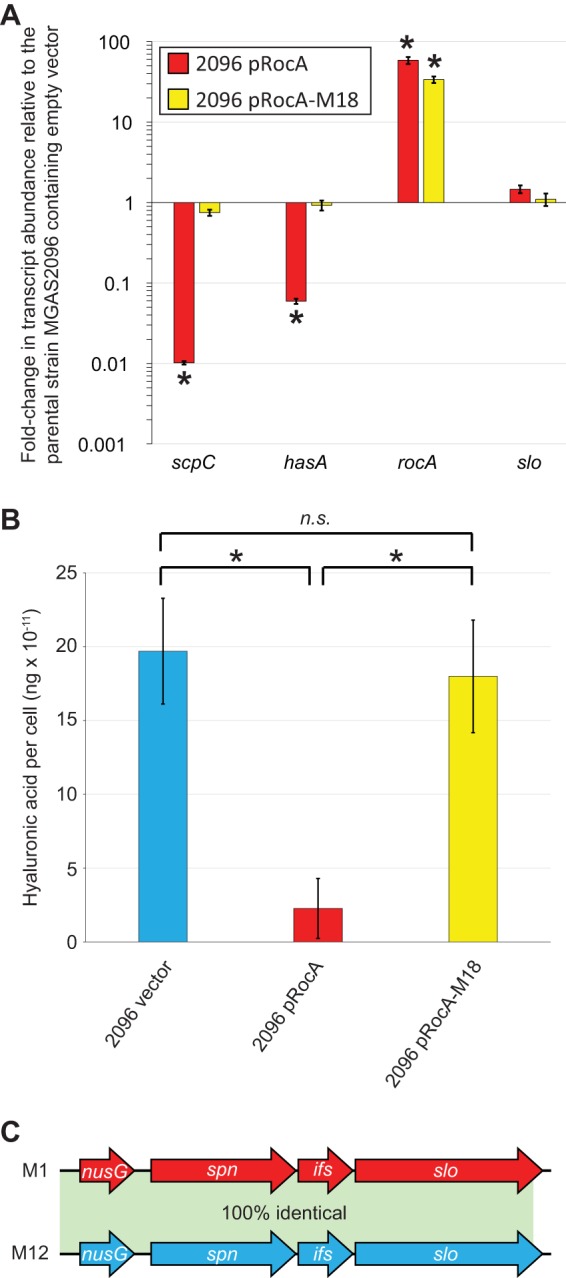
Exogenously expressed RocA complements the *rocA* mutation of strain MGAS2096. (A) TaqMan-based quantitative RT-PCR analysis comparing MGAS2096-based derivatives containing empty vector (pDCBB), a plasmid expressing a functional *rocA* allele (pRocA), or a plasmid expressing the nonfunctional *rocA* allele from M18 GAS (pRocA-M18). The abundance of the indicated mRNAs was determined from duplicate exponential-phase GAS cultures, run in duplicate. Values shown are means ± standard deviations (error bars). Values that are statistically significantly different (*P < *0.05 by Wilcoxon signed rank test) from the values for the isolates containing empty vector are indicated by an asterisk. (B) Assay of capsule expression. Exponential-phase cultures of the indicated GAS strains were analyzed for levels of the hyaluronic acid capsule. The experiment was performed on three occasions, using duplicate cultures of each strain in each experiment, with mean ± standard deviation values shown. *P < *0.01 (via overall analysis of variance [ANOVA]). The values for individual strains were compared by Tukey’s multiple-comparison test and indicated as follows: *, *P* < 0.05; *n.s.*, not significant. (C) Schematic showing that *slo* and the two promoters that drive *slo* transcription, located upstream of *spn* and *nusG*, are identical between the tested M12 and M1 GAS strains.

To confirm that strain MGAS2096 has a *rocA* allele that produces functional protein, we performed complementation analysis. Three plasmids were independently transformed into MGAS2096, the empty vector, pRocA (the vector containing a functional *rocA* allele), and pRocA-M18 (the vector containing a mutant *rocA* allele as found in serotype M18 isolates [11]). As expected for a *rocA* mutant strain, the introduction of a functional *rocA* allele, but not a mutant allele or the empty vector, dramatically reduced the abundance of *scpC* and *hasA* transcripts (Fig. 3A). The reduction in *hasA* transcript levels in strain 2096 pRocA resulted in a concomitant reduction in capsule expression (Fig. 3B). In combination with previously published data, which show that overexpressing functional RocA has only regulatory consequences in a *rocA* mutant strain background (35), these data confirm that MGAS2096 harbors a mutant *rocA* allele. Interestingly, *slo* transcript levels are unchanged in strain 2096 pRocA, despite the well-characterized repression of *slo* transcription by CovR/S and RocA in other strains/serotypes (14, 38). We propose that this regulatory difference is a consequence of strain- or serotype-specific regulation. Note however, that these differences cannot be attributed to variations in the promoter regions that produce *slo* transcripts, as they are identical between the tested M1 and M12 strains (Fig. 3C) (48, 49).

### Enhanced survival in human blood *ex vivo* following complementation of the MGAS2096 *rocA* mutation.

**FIG 4:**
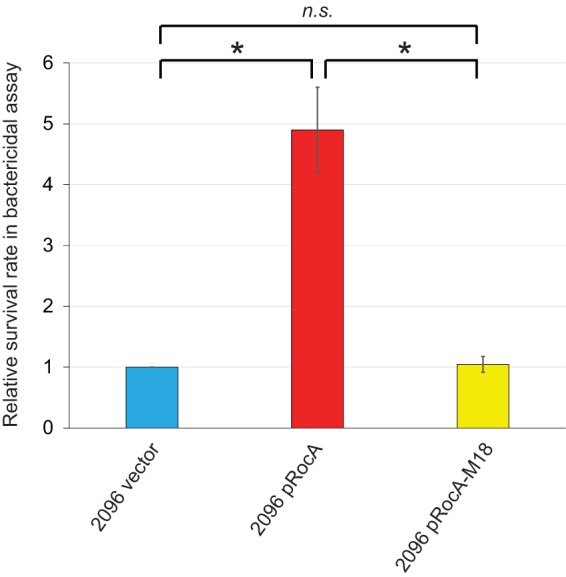
RocA enhances the ability of strain MGAS2096 to survive and proliferate in blood. Bactericidal assays were performed with heparinized whole human blood and the three indicated GAS strains. The experiment was performed in triplicate with the combined data shown. The data are presented as the GAS survival rate relative to that observed for the parental isolate MGAS2096 containing empty vector. Error bars show standard deviations. *P < *0.01 (via overall ANOVA). Individual strains were compared by Tukey’s multiple-comparison test and indicated as follows: *, *P* < 0.05; *n.s.,* not significant.

In all GAS strains tested thus far, *rocA* mutation resulted in an enhanced ability to survive and proliferate in nonimmune whole human blood (11, 35, 38). We tested whether the same phenotype is observed between our plasmid-containing MGAS2096-based strains and, to our surprise, identified the opposite phenotype (Fig. 4). Complementation of *rocA* in strain MGAS2096 increased, not decreased, the ability of this strain to survive in a Lancefield bactericidal assay. A molecular explanation for this variant phenotype is currently lacking, but we hypothesize that the observed variation by which genes are regulated by RocA in this strain or serotype is a contributing factor.

### *rocA* is transcribed via two distinct transcript forms.

**FIG 5:**
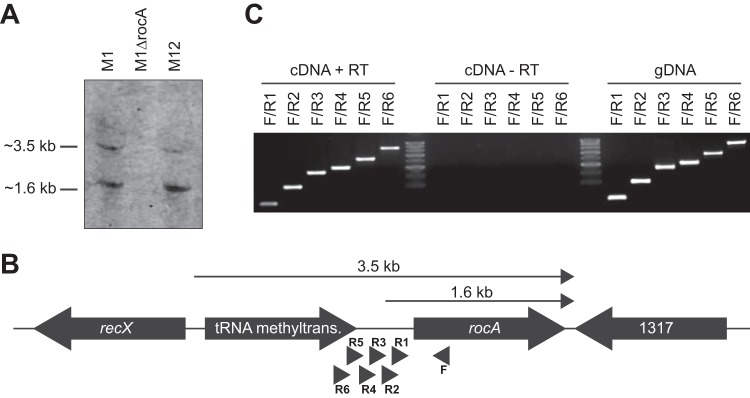
Transcription of *rocA* occurs through its own promoter and via cotranscription with the upstream tRNA methyltransferase-encoding gene. (A) Northern blot analysis showing the presence of two *rocA*-containing transcripts. (B) Schematic showing the likely locations of the observed 1.6-kb and 3.5-kb transcripts. (C) RT-PCR analysis is consistent with a subset of *rocA*-containing transcripts also including the upstream tRNA methyltransferase-encoding gene. The relative locations of the primers used in this analysis are shown in panel B. Genomic DNA (gDNA) was used as the template for a positive control for the PCRs. For a negative control, we used a no reverse transcriptase cDNA synthesis reaction (cDNA – RT) (this controls against contaminating gDNA in the isolated RNA). The cDNA + RT data represent the test data.

As RocA is a major regulator of GAS virulence, we thought it prudent to characterize how the *rocA* gene itself is regulated. In particular, we investigated the size of *rocA*-containing transcripts to provide insight into how *rocA* is transcribed, and we also investigated the relative abundance of *rocA*-containing transcripts to determine whether any differences exist between strain MGAS2096 and the serotype M1 strain MGAS2221. Northern blot analysis identified the presence of two distinct *rocA*-containing transcripts, one ∼3.5 kb and one ∼1.6 kb (Fig. 5A). These two transcripts were present in both MGAS2221 and MGAS2096, indicating that large-scale differential *rocA* transcription does not occur between these isolates under the conditions assayed. The ∼1.6-kb transcript is consistent with the expected size of the *rocA* gene by itself, whereas the ∼3.5-kb transcript is consistent with it consisting of both *rocA* and the upstream tRNA methyltransferase-encoding gene (Fig. 5B). To confirm that there is transcriptional read-through from the tRNA methyltransferase-encoding gene into *rocA*, we performed reverse transcription-PCR (RT-PCR) analysis. A single forward primer (F) embedded within *rocA* was paired with six different reverse primers (R1 to R6) located with increasing distance from *rocA* (Fig. 5B). All primer pairs resulted in a product, including the F/R6 reaction where primer R6 is located within the upstream tRNA methyltransferase-encoding gene (Fig. 5C). Thus, not only does *rocA* have its own promoter but this gene is also cotranscribed with the upstream gene.

## DISCUSSION

The population of bacteria causing any given infection is most often heterogeneous, and in part this heterogeneity is a consequence of phenotypic variants being selected for during infection (14, 23, 32, 36, 50–52). Such phenotypic variants can have enhanced abilities to circumvent the host immune response (14), altered adherence properties (53), altered metabolism/growth profiles (54), altered tissue tropism (55), altered antibiotic resistance profiles (56), etc. Thus, the whole community of bacteria present during infection needs to be considered when investigating and treating such infections. Phenotypic variation often occurs as a consequence of gene mutation, with the mutation of regulatory genes (i.e., those encoding regulators of gene transcription) being particularly prevalent (57, 58). Here, we present data showing that the serotype M12 GAS strain MGAS2096, which was whole genome sequenced in 2007, has an inactivating mutation in *rocA*. As RocA encodes an accessory protein to the CovR/S two-component regulatory system, the mutation of *rocA* in MGAS2096 dramatically alters virulence factor expression. In contrast to previous RocA studies, *rocA* mutation reduces rather than enhances the ability of this strain to survive and proliferate in human blood.

*rocA* mutant strains have been described across several different GAS serotypes, but the distribution of such mutants varies. For example, serotype M3 and M18 isolates are exclusively *rocA* mutants (i.e., there are no M3 or M18 GAS isolates that produce a functional RocA) (10, 11), but only select serotype M1 and M89 isolates have *rocA* mutations (12, 34). Similar to the well-characterized selection of *covR* and *covS* mutant strains (14, 23), most likely due to the higher expression of immunomodulatory virulence factors by *covR* and *covS* mutants, *rocA* mutations are also selected for during invasive GAS infections (34, 36, 37). Given that CovR, CovS, and RocA are all required for significant repressive activity by CovR, the mutation of any one of the encoding genes result in similar, but not identical, phenotypes (14, 15, 36). The *rocA* inactivating mutation in MGAS2096 is unique to this strain, it is not present in the *rocA* alleles of MGAS9429, TJ11-001 (an M12 isolate from China) (59), HKU360 (an M12 isolate from Hong Kong) (60), or any other thus-far described GAS isolate. Consequently, serotype M12 GAS isolates are not exclusively *rocA* mutants, as M3 and M18 isolates are, rather they are likely similar to M1 and M89 isolates, where the majority (e.g., MGAS9429, TJ11-001, HKU360) have a functional *rocA* gene but mutant derivatives (e.g., MGAS2096) can arise during infection. Thus, we hypothesize that the infected patient from which MGAS2096 was isolated harbored a mixture of GAS, an original infecting strain that had a functional *rocA* gene and a *rocA* mutant derivative that was selected for at some point during infection (represented by MGAS2096).

The *rocA* mutation in MGAS2096 consists of an expansion of a mononucleotide VNTR, with five T nucleotides in a wild-type strain (e.g., MGAS9429) and six in MGAS2096 (Fig. 1A). The addition of a single T nucleotide likely arose as a consequence of slipped-strand mispairing during DNA replication, as has been hypothesized for alterations in repeat copy number for other VNTRs (10, 12, 14). Previously, we have shown that overexpressing only the N-terminal half of RocA, which contains the six transmembrane domains but not the DHp or HATPase domains (Fig. 1B), is sufficient to complement a *rocA* mutant strain (35). While the mechanism by which RocA enhances the abundance of phosphorylated CovR is unknown, the lack of a requirement of the C-terminal half of RocA is consistent with other data suggestive of RocA being a pseudokinase (35, 61). We are currently testing the working hypothesis that RocA and CovS interact via their transmembrane domains and that this interaction enhances the kinase activity of CovS toward CovR. The RocA expressed in strain MGAS2096 is truncated within the first transmembrane domain (Fig. 1B), and since all six transmembrane domains are required for activity, even when overexpressed (35), this explains the lack of RocA activity in this strain.

That RocA regulates virulence factor expression in M12 GAS was expected given previous publications regarding the function of this protein (11, 35, 38, 61). However, there appear to be variations in exactly which virulence factors are under the regulatory control of RocA in MGAS2096 relative to previously studied isolates, none of which were serotype M12 isolates. Transcript abundance from the *slo* gene is negatively regulated by RocA ∼10-fold or higher in serotype M1, M3, and M89 strains (12, 35, 38) but was unchanged following *rocA* complementation in MGAS2096 (Fig. 3A). Thus, the assortment of genes regulated by RocA appears to be flexible. However, as MGAS2096 is the only M12 strain studied in this regard, it is unknown whether the lack of RocA-mediated regulatory activity toward *slo* transcription is serotype specific or whether it is unique to MGAS2096. The lack of *slo* regulation by RocA in MGAS2096 is not a consequence of mutations occurring within the promoter regions that drive *slo* transcription, as these regions are identical between both M12 isolates and the M1 isolate studied here (Fig. 3C). Note that the absence of genetic alterations (i.e., single nucleotide polymorphisms [SNPs]) between these genes in M1 and M12 isolates is a consequence of a 36-kb recombinational replacement that occurred in an ancestral isolate of contemporary serotype M1 strains. This recombinational replacement replaced the previous M1 alleles of these genes with the M12 alleles, resulting in increased streptolysin O expression by contemporary M1 isolates (62, 63).

We hypothesize that variation in which genes are regulated by RocA is behind the divergent survival phenotype seen for MGAS2096 and its complemented derivative in a bactericidal assay (Fig. 4). While *rocA* mutants have higher survival rates in human blood *ex vivo* than isolates with functional *rocA* genes in M1, M3, and M18 strain backgrounds (11, 35, 38), the reverse is true for MGAS2096 and its *rocA*-complemented derivative. Why the opposite phenotype is observed for these strains is unknown and is surprising given the previous bactericidal data and the fact that the *rocA*-complemented MGAS2096 derivative has reduced capsule expression (Fig. 3B), which normally correlates with reduced survival in blood. Future research looking at the transcriptomes of these isolates, rather than select mRNAs (Fig. 3A), may shed light on the molecular basis of this phenotype. Obviously, survival in blood is only one phenotype, and it may be the case that the *rocA* mutation of MGAS2096 was selected for due to some other, as-yet-uncharacterized, phenotype. It should also be noted that the isolation of GAS from patients with APSGN, as was the case for MGAS2096, is rare, as in most cases the infection has cleared prior to clinical disease (39). Perhaps *rocA* mutation assisted in prolonging the infection until after the point where symptoms of glomerulonephritis appeared.

We identified that *rocA* is transcribed as part of two different transcripts. One transcript originates from a promoter upstream oftranscript originates from a promoter upstream of *rocA* and is monocistronic (Fig. 5). The second transcript originates from a promoter upstream of a tRNA methyltransferase-encoding gene which is located upstream of *rocA* (Fig. 5) and is polycistronic. It is tempting to speculate that there are regulatory consequences to the relative abundances of the large (tRNA methyltransferase and *rocA*) and small (*rocA* only) transcripts, but this has yet to be investigated.

In conclusion, we have identified a serotype M12 GAS strain that has a naturally occurring null mutation in *rocA*, resulting in a divergent transcriptional and phenotypic profile. The data support a key regulatory role for RocA and uncover the existence of serotype- or strain-specific variation in the targets of RocA-mediated regulation. Given the virulence-regulating role of RocA, further study of this protein, including the mechanism by which it positively regulates the activity of the CovR/S two-component system, is warranted.

## MATERIALS AND METHODS

### Bacterial strains and growth conditions.

The serotype M12 clinical GAS isolates MGAS2096 and MGAS9429 were used in this study (39). Information about these strains, and others created and/or used in this study, is present within Table 1. GAS were grown in Todd-Hewitt broth containing 0.2% yeast extract (THY broth). Chloramphenicol was added when needed, to a final concentration of 4 µg/ml.

**TABLE 1:** GAS strains used in this study

GAS strain	Description	Reference
MGAS2096	A serotype M12 GAS strain that was isolated from a patient with acute poststreptococcal glomerulonephritis in 1960. Contains a 1-bp insertion in *rocA*	39
MGAS9429	A serotype M12 GAS strain that was isolated from a pediatric patient with pharyngitis in 2001. Contains a functional *rocA* allele	39
MGAS2221	A serotype M1 GAS strain that has been extensively characterized	14
2221ΔrocA	MGAS2221 derivative in which the *rocA* gene has been replaced with a spectinomycin resistance cassette	38
2096 pDCBB	MGAS2096 derivative containing the chloramphenicol-resistant empty vector pDCBB	This study
2096 pRocA	MGAS2096 derivative containing the chloramphenicol-resistant pRocA which expresses functional RocA (as found in the M1 GAS strain MGAS2221)	This study
2096 pRocA-M18	MGAS2096 derivative containing the chloramphenicol-resistant pRocA-M18 which expresses a nonfunctional RocA (as found in M18 GAS)	This study

### Creation of MGAS2096 derivative strains.

Three plasmids were individually transformed into MGAS2096, creating three derivatives. Strain “2096 vector” is MGAS2096 containing the empty vector pDCBB, which is a derivative of pDC123 in which the *phoZ* gene has been deleted (64). Strain “2096 pRocA” is MGAS2096 containing the pDCBB derivative plasmid pRocA (35), which contains the functional *rocA* allele from GAS strain MGAS2221. Strain “2096 pRocA-M18” is MGAS2096 containing the pDCBB derivative plasmid pRocA-M18 (35), which contains the nonfunctional *rocA* allele from the serotype M18 GAS strain MGAS8232.

### Hyaluronic acid capsule assays.

Hyaluronic acid capsule assays were performed as we have previously described (65). Briefly, GAS strains were pelleted by centrifugation and resuspended in 500 μl of water, and serial dilutions were performed to ensure equivalent numbers of CFU for all of the strains. To remove the capsule from the bacteria, 400 μl of each suspension was placed in a 2-ml screw-cap tube containing 1 ml of chloroform and run in a FastPrep machine at speed 4.5 for 1 min. After cooling on ice for 1 min, the samples were reprocessed in the FastPrep machine before centrifugation at 13,000 × *g* for 10 min. The aqueous phase was transferred to a clean tube, and the hyaluronic acid content was determined using an enzyme-linked immunosorbent assay (ELISA) kit (Corgenix) in accordance with the manufacturer’s instructions.

### Isolation of secreted GAS proteins and Western blot analyses.

Supernatant proteins from exponential-phase THY broth GAS cultures were concentrated by ethanol precipitation and resuspended in SDS-PAGE buffer at 1/20th the original volume. Equal concentrations of each protein sample were separated on 12% Tris-HCl gels before transferring to membrane and using in Western blot analysis with a custom rabbit anti-SKA polyclonal antibody (made by Paciﬁc Immunology Inc.) or a commercial rabbit anti-SLO polyclonal antibody (American Research Products Inc.). After washing, Alexa Fluor 680-labeled donkey anti-rabbit IgG secondary antibody (Molecular Probes) was used (1:10 000 dilution), and the ﬂuorescent signal was detected using a Li-Cor Odyssey near-infrared imaging system. An identical gel was stained with Coomassie blue to serve as a loading control.

### Quantitative RT-PCR analyses.

RNA samples from triplicate exponential-phase cultures of each GAS strain under investigation were isolated and converted into cDNA as previously described (14). TaqMan primers and probes for the genes of interest and the internal control gene *proS* are shown in Table 2. Transcript levels were determined using the ΔΔ*C_T_* method.

**TABLE 2:** Primers and probes used in this study

Primer or probe	Sequence	Description
F (UNR511)	GAATGAAATGGTCTGGAAAGAAAG	Forward primer used in the RT-PCR analysis of Fig. 5
R1 (UNR525)	GGATAAATGTTAGAAGATTTTC	Reverse primer used in the RT-PCR analysis of Fig. 5
R2 (UNR513)	CTGTTAGAATGACAGAACTTATG	Reverse primer used in the RT-PCR analysis of Fig. 5
R3 (UNR512)	GTAGGCTGTGTGAGTCTTTATG	Reverse primer used in the RT-PCR analysis of Fig. 5
R4 (UNR529)	GATATAGAGGATTTATCCTGATTTAATC	Reverse primer used in the RT-PCR analysis of Fig. 5
R5 (UNR531)	GAGCAAGTACACACAGACAATAT	Reverse primer used in the RT-PCR analysis of Fig. 5
R6 (UNR534)	GTGAAGTTACAAAAACGTGTATG	Reverse primer used in the RT-PCR analysis of Fig. 5
UNR342	CGTTATGTAAAACAAAACTCTATTGAG	Used with UNR343 to create a probe for the Northern blot shown in Fig. 5
UNR343	TCAGTCAGGCTTAGCTATTTCTATTAACTG	Used with UNR342 to create a probe for the Northern blot shown in Fig. 5
proS.UTMF	TACCACTGGCAAATCGTACC	TaqMan primer to detect *proS*
proS.UTMR	CATTTCAACAGCACCGATCT	TaqMan primer to detect *proS*
proS.UTMP	CACGCATGATGGTCTTGAATTTCTCA	TaqMan probe to detect *proS*
grab.TMF	GCATCAGTATTAGTCGGTTCAACAGT	TaqMan primer to detect *grab*
grab.TMR	GGTTCCGCCATTTGGAATAA	TaqMan primer to detect *grab*
grab.TMP	TGTTGACTCACCTATCGAACAGCCTCGA	TaqMan probe to detect *grab*
scpCTMF	AAGGAGCTTGGGACAAGGGATA	TaqMan primer to detect *scpC*
scpCTMR	TGATGGGCCGGATCGA	TaqMan primer to detect *scpC*
scpCTMP	CAATAACTGCGACAACCTTGCCTTGTCCT	TaqMan probe to detect *scpC*
hasATMF	ATGATCGATGTTTAACAAATTATGCTATTG	TaqMan primer to detect *hasA*
hasATMR	TTAAATAACTTTTTAATTGGAAAGGTACATCAG	TaqMan primer to detect *hasA*
hasATMP	ACGCACTGTCTACCAATCAACAGCTAGATGTG	TaqMan probe to detect *hasA*
rocATMF	AGGGCTATAAGCGCAAAGAA	TaqMan primer to detect *rocA*
rocATMR	GGCTTTCTTTCCAGACCATT	TaqMan primer to detect *rocA*
rocATMP	TGAGCCAACATCACAACATCAAGAATG	TaqMan probe to detect *rocA*
sloTMF	GACCTTTAAAGAGTTGCAACGAAAA	TaqMan primer to detect *slo*
sloTMR	GACCATAAGCTACGTTACTCACAAAGA	TaqMan primer to detect *slo*
sloTMP	TGTCAGCAATGAAGCCCCGCC	TaqMan probe to detect *slo*

### Lancefield bactericidal assays.

To test the ability of individual GAS strains to survive in human blood, we performed modified Lancefield bactericidal assays. Cultures of each strain were grown to early exponential phase (an optical density at 600 nm [OD_600_] between 0.15 and 0.20). Each GAS culture was diluted to 10^−4^ in sterile phosphate-buffered saline, and 450 µl of whole heparinized blood was added to 50 µl dilute culture. These mixtures were then incubated for 3 h at 37°C with end-over-end rotation. Fifty microliters of each inoculum was simultaneously plated on blood agar plates to allow enumeration on the next day. Following incubation, the GAS-blood cultures were diluted and plated on blood agar plates. All samples were incubated overnight at 37°C in a 5% CO_2_ atmosphere. The number of CFU was calculated, and the data were presented as the rate of survival relative to that of the empty-vector-containing strain after performing the calculation [(number of surviving CFU/initial number of CFU) × 100]. Blood samples used in this assay were obtained in accordance with the guidelines set forth in a protocol approved by the University of Nevada, Reno (UNR) Institutional Review Board (IRB).

### Northern blot analysis.

Total RNA was isolated from exponential-phase cultures of the serotype M1 strain MGAS2221, a *rocA* mutant derivative of MGAS2221 (strain 2221ΔrocA), and the serotype M12 strain MGAS2096. The RNA isolation procedure used was identical to that previously described (14). For each strain, 12 µg of RNA was loaded onto a 0.8% agarose gel made with 1× morpholinepropanesulfonic acid (MOPS). After electrophoresis and RNA transfer, the blot was prehybridized in Ultra Hyb buffer (Ambion). The blot was probed overnight in Ultra Hyb buffer after the addition of a biotinylated probe, created via PCR using the primers UNR342 and UNR343 (Table 2) in reaction mixtures that included biotin-16-dUTP (Roche). The following morning, the blot was washed, blocked, treated with streptavidin IRDye 680 (Li-Cor), washed again, and visualized on a Li-Cor Odyssey near-infrared imaging system.

### RT-PCR analysis.

Total RNA was isolated from strain MGAS2221 and used to generate cDNA as previously described (14). The generated cDNA was used in RT-PCR analysis to verify that *rocA* is cotranscribed with the upstream gene. A single forward primer was designed located within *rocA* (primer F in Fig. 5B). Six reverse primers were designed (primers R1 through R6 in Fig. 5B) for use in conjunction with the forward primer, and these primers are located at increasing distance from *rocA* (with R6 being wholly located within the upstream tRNA methyltransferase-encoding gene). The PCRs were set up, separated on a 1% agarose gel, and photographed. PCRs using genomic DNA (gDNA) as the template were used as positive controls, while reactions using mock cDNA synthesis reactions as the template (where no reverse transcriptase was added to the reaction; cDNA-RT) were used as controls against gDNA contamination.
